# Emergency medical system utilization among patients with end-stage kidney disease during the COVID-19 pandemic: a retrospective cohort study in South Korea

**DOI:** 10.3389/fpubh.2026.1687521

**Published:** 2026-04-07

**Authors:** Kyung Won Kim, Hyunjin Cho, Hye Eun Yoon, Young-Ki Lee, Kyung Don Yoo, AJin Cho

**Affiliations:** 1 Department of Internal Medicine, Konkuk University Medical Center, Konkuk University School of Medicine, Seoul, Republic of Korea; 2 Department of Internal Medicine, Seoul St Mary’s Hospital, College of Medicine, The Catholic University of Korea, Seoul, Republic of Korea; 3 Transplantation Research Center, College of Medicine, The Catholic University of Korea, Seoul, Republic of Korea; 4 Department of Internal Medicine, Kangnam Sacred Heart Hospital, Seoul, Republic of Korea; 5 Hallym Kidney Research Institute, Hallym University College of Medicine, Seoul, Republic of Korea; 6 Division of Nephrology, Department of Internal Medicine, Ulsan University Hospital, University of Ulsan College of Medicine, Ulsan, Republic of Korea; 7 Basic-Clinical Convergence Research Institute, University of Ulsan, Ulsan, Republic of Korea; 8 Department of Medical Science Convergence, Graduate School of Medical Science, University of Ulsan, Ulsan, Republic of Korea

**Keywords:** chronic kidney disease, COVID-19, emergency department use, hemodialysis, SARS-CoV

## Abstract

**Introduction:**

Patients with end-stage kidney disease (ESKD) are particularly vulnerable to adverse outcomes from coronavirus disease 2019 (COVID-19). We aimed to compare emergency department (ED) utilization patterns and clinical outcomes among patients with ESKD before and during the COVID-19 pandemic to inform future healthcare planning.

**Methods:**

We analyzed data from the National Emergency Department Information System (NEDIS), a nationwide database of ED visits in South Korea. Adult patients with ESKD who visited the ED between January 1, 2018, and May 31, 2021, were included. ED visits were categorized into pre- COVID-19 (2018-2019) and COVID-19 (2020-2021) periods based on visit date. Trends in ED visit frequency, presenting causes, clinical severity, hospitalization, and short-term mortality were compared using descriptive statistics and multi-variate logistic regression.

**Results:**

A total of 159,456 ED visits were analyzed (76,277 in 2018-2019; 83,179 in 2020-2021). Compared with the pre-pandemic period, ED visits during the COVID-19 period increased and were associated with higher odds of hospitalization [adjusted odds ratio (aOR), 1.054; 95% confidence interval (CI), 1.031–1.079] and mortality (aOR, 1.052; 95% CI, 1.007–1.099). This trend was most notable among patients presenting with severe conditions. Vascular access complications were the leading cause of ED visits during the COVID-19 period. Patients with severe illness were more likely to visit high-level hospitals and had higher rates of hospitalization and in-hospital mortality during the pandemic.

**Conclusion:**

The study found that patients with ESKD experienced an increase in ED visits during the COVID-19 pandemic, along with a higher risk of hospitalization and mortality; however, the distribution of patients across hospital service levels improved during the outbreak. These findings can inform preparedness strategies for future public health emergencies.

## Introduction

The onset of coronavirus disease 2019 (COVID-19), initially identified in Wuhan, China, in late 2019, resulted in unprecedented global health disruptions and was officially declared a pandemic by the World Health Organization on March 11, 2020 (1, 2). As the disease spread rapidly worldwide, public health interventions including lockdowns, social distancing, and the reallocation of healthcare resources significantly impacted patterns of healthcare utilization and restricted access to routine medical services (3, 4). Although efforts were made to maintain dialysis care continuity, patients with end-stage kidney disease (ESKD) experienced disruptions in both routine and emergency medical services during the pandemic (5–7).

ESKD is associated with significant morbidity and high burden of acute care utilization, including frequent emergency department (ED) visits (8, 9). These visits are often necessitated by complications such as fluid overload, electrolyte imbalance, vascular access issues, and infections, reflecting the vulnerable clinical status of this patient population (9, 10). In addition to dialysis-related complications, patients with ESKD have a higher risk of cardiovascular and cerebrovascular events. Consequently, hospitalization and mortality rates following ED visits are substantially higher in this population than in the general population (11). ED visits by patients with ESKD were characterized by significantly higher hospitalization (66.7%) and in-hospital mortality rates (9.4%) than those of patients without chronic kidney disease (21.0% and 5.1%, respectively) (12). The risk factors associated with poor outcomes included older age, male sex, inter-facility transfer, high triage acuity, and prolonged ED stay. These findings highlight the clinical vulnerability of patients with ESKD. Given the critical need for uninterrupted medical care in this population, any delays or obstacles to emergency services may lead to adverse clinical consequences, including increased disease severity at presentation and higher short-term mortality.

Despite these concerns, there is limited evidence on how ED utilization patterns and clinical outcomes among patients with ESKD were affected before and after the onset of the COVID-19 pandemic. A comprehensive understanding of these trends is critical to improve emergency preparedness, continuity of care, and policy planning for dialysis-dependent patients during future public health crises. We aimed to evaluate temporal changes in ED utilization and clinical outcomes among patients with ESKD in South Korea before and during the COVID-19 pandemic using nationwide population-based data.

## Methods

### Study design and data source

We conducted a comprehensive nationwide retrospective cohort study using data from the National Emergency Department Information System (NEDIS) from January 1, 2018, to December 31, 2021. On January 28, 2020, South Korea was declared to be in an “orange” infectious disease crisis phase after the first case of COVID-19 was detected on January 20, 2020. Local health, district medical, and emergency medical centers were designated as COVID-19 screening centers, where patients were screened for fever or respiratory symptoms, recommended COVID-19 testing, and not immediately referred to a hospital. The present study investigated temporal changes in the frequency of ED use, variations in presenting causes and clinical severity, and differences in hospitalization and short-term mortality outcomes during the COVID-19 pandemic (from January 1, 2020, to December 31, 2021) and the preceding period (from January 1, 2018, to December 31, 2019).

The government established the NEDIS in 2003 as an emergency medical monitoring system linking various types of public information to build a single database; it was designed to identify the operational status of the emergency medical system regarding emergency patient occurrence, transportation, treatment, and discharge. NEDIS laid the foundation for building an advanced emergency medical system and providing basic data for research and policy formulation for emergency medical care (13).

The study was conducted in accordance with the principles of the Declaration of Helsinki. The database was fully anonymized, and the requirement for informed consent was waived by the Ethics Committee of the Ulsan University Hospital Institutional Review Board (IRB No. UUH2024-01-017 and UUH2023-10-034).

### Study population

**Figure 1:**
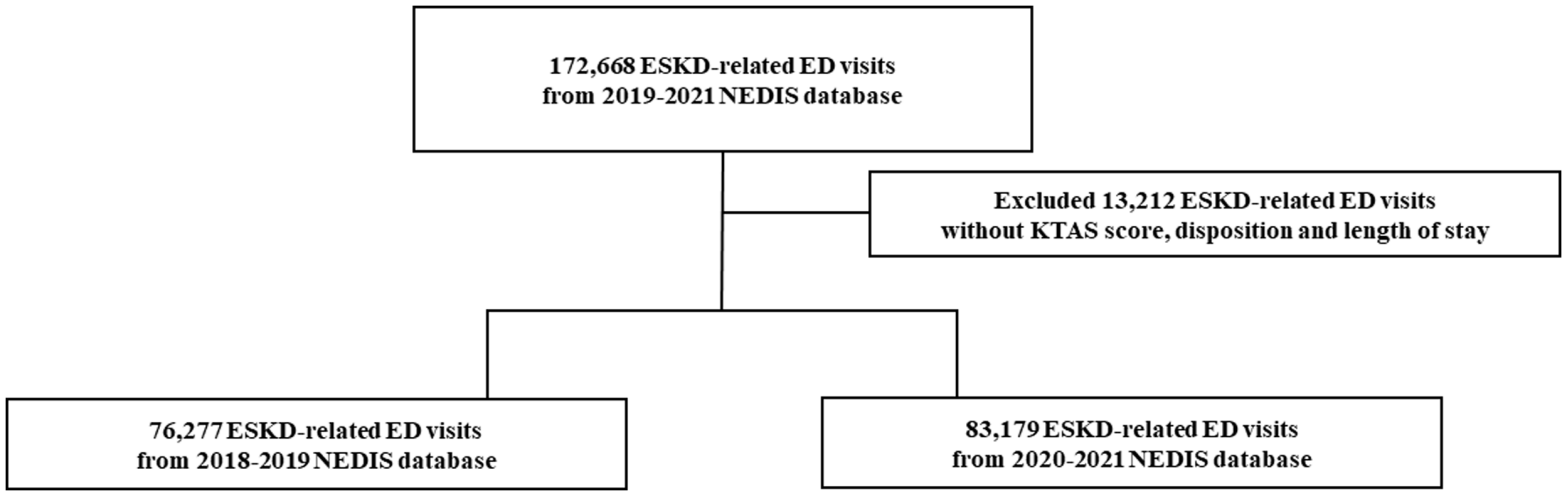
Flowchart of the enrolled study population.

ED visits by patients with ESKD were selected using the modified version of the International Classification of Diseases, 10th Revision (ICD-10) diagnosis codes. This study included ED visits of patients aged ≥20 years with a primary or secondary ICD-10 code diagnosis of N18.5. Initially, 172,668 ESKD-related ED visits were included; 13,212 visits with missing data were excluded, resulting in a total of 76,277 ESKD-related ED visits in 2018-2019 and 83,179 ED visits in 2020-2021 (Figure 1).

### Variables

Demographic characteristics such as age and sex, insurance status, hospital level, route of arrival (direct visits, transfers from another hospital or outpatient clinic, others, or unknown), transportation (ambulance, non-ambulance, or unknown), and length of stay were included. The Korean Triage System (KTAS) is a triage system used to categorize patients during ED visits. It was developed through the Korean Emergency Patient Severity Classification System Standardization Study from 2012 to 2015 and has been implemented in all EDs in Korea since 2016 (14, 15). The KTAS consists of 5 levels based on symptoms and vital signs: 1. Resuscitation; 2. Emergency; 3. Urgent; 4. Less urgent; and 5. Non-urgent. Hospital service levels were classified by the Ministry of Health and Welfare into 3 levels depending on their size and function: Level 1, Regional Emergency Medical Center (REMC); Level 2, Community Emergency Medical Center (CEMC); and Level 3, Community Emergency Medical Institute (CEMI).

### Statistical analysis

Data are presented as frequencies (percentages) and means [standard deviations (SD)]. We compared the baseline characteristics of ESKD-related ED visits between 2018-2019 and 2020-2021. We then assessed differences in ED visit characteristics between the two time periods according to hospital service level. Categorical variables were compared using the chi-square test, and continuous variables were analyzed using t-tests or analysis of variance. Multivariate logistic regression analysis was used to investigate risk factors for hospitalization and in-hospital mortality after an ED visit, as well as the impact of COVID-19 on clinical outcomes among patients with ESKD. Age, sex, insurance status, hospital service level, route of arrival, KTAS classification, transportation, and length of stay were adjusted. All tests were two-tailed, and a *p*-value <0.05 was considered significant. All data preparation and statistical analyses were performed using R version 4.0.5 (R Foundation for Statistical Computing, Vienna, Austria)^1^.

## Results

### Characteristics of ESKD-related ED visits before and during COVID-19

ED visits during 2020-2021 accounted for 52.2% of the total 159,456 ESKD-related ED visits (Table 1). During the COVID-19 outbreak, patients with ESKD visited the ED more frequently than in previous periods. ED visits during COVID-19 included a higher proportion of patients aged ≥80 years. Differences in sex and insurance status were not statistically significant. During COVID-19, patients with ESKD were more likely to be transferred from outpatient clinics, use ambulances, and be in serious KTAS conditions (Levels 1–3).

**Table 1:** Baseline characteristics of ESKD patients who visited the emergency department before and during COVID-19.

Variables	Total	ESKD 2018-2019	ESKD 2020-2021	*p*-value
Number of emergency department visits	159,456	76,277	83,179	
Age (years)				<0.001
20–29	1,818 (1.14)	859 (1.13)	959 (1.15)	
30–39	5,343 (3.35)	2,495 (3.27)	2,848 (3.42)	
40–49	13,041 (8.18)	6,684 (8.76)	6,357 (7.64)	
50–59	28,992 (18.18)	14,656 (19.21)	14,336 (17.24)	
60–69	41,024 (25.73)	19,599 (25.69)	21,425 (25.76)	
70–79	42,165 (26.44)	20,254 (26.55)	21,911 (26.34)	
≥80	27,073 (16.98)	11,730 (15.38)	15,343 (18.45)	
Sex				0.001
Men	91,576 (57.43)	43,478 (57.00)	48,098 (57.82)	
Women	67,880 (42.57)	32,799 (43.00)	35,081 (42.18)	
Insurance status				<0.001
National health insurance	120,661 (75.67)	57,674 (75.61)	62,987 (75.72)	
Medical aid	37,133 (23.29)	17,908 (23.48)	19,225 (23.11)	
Others	988 (0.62)	428 (0.56)	560 (0.67)	
Uninsured	367 (0.23)	200 (0.26)	167 (0.20)	
Unknown	307 (0.19)	67 (0.09)	240 (0.29)	
Hospital service level				<0.001
I (REMC)	62,728 (39.34)	29,994 (39.32)	32,734 (39.35)	
II (CEMC)	84,281 (52.86)	41,750 (54.73)	42,531 (51.13)	
III (CEMI)	12,447 (7.81)	4,533 (5.94)	7,914 (9.51)	
Route of arrival				<0.001
Direct visit	105,588 (66.22)	50,179 (65.79)	55,409 (66.61)	
Transferred from other hospital	40,377 (25.32)	20,123 (26.38)	20,254 (24.35)	
Transferred from outpatient clinic	13,366 (8.38)	5,908 (7.75)	7,458 (8.97)	
Other	123 (0.08)	66 (0.09)	57 (0.07)	
Unknown	2 (0.00)	1 (0.00)	1 (0.00)	
Transportation				<0.001
119 ambulance	41,467 (26.01)	19,111 (25.05)	22,356 (26.88)	
Other ambulance	4,191 (2.63)	2,172 (2.85)	2,019 (2.43)	
Non-ambulance	18,404 (11.54)	9,060 (11.88)	9,344 (11.23)	
Unknown	95,394 (59.82)	45,934 (60.22)	49,460 (59.46)	
KTAS classification				<0.001
1. Resuscitation	5,928 (3.72)	2,747 (3.60)	3,181 (3.82)	
2. Emergent	23,696 (14.86)	10,913 (14.31)	12,783 (15.37)	
3. Urgent	89,205 (55.94)	41,980 (55.04)	47,225 (56.78)	
4. Less urgent	28,790 (18.06)	14,697 (19.27)	14,093 (16.94)	
5. Non urgent	11,837 (7.42)	5,940 (7.79)	5,897 (7.09)	
Length of stay	8.11 ± 10.23	8.15 ± 10.90	8.07 (9.58)	0.081
Disposition				<0.001
Discharge home	49,519 (31.05)	24,612 (32.27)	24,907 (29.94)	
Transfer to other hospital	2,871 (1.80)	1,374 (1.80)	1,497 (1.80)	
Admission				
General ward	79,325 (49.75)	37,664 (49.38)	41,661 (50.09)	
ICU	26,778 (16.79)	12,167 (15.95)	14,611 (17.57)	
Other	17 (0.01)	6 (0.01)	11 (0.01)	
Died in ED	688 (0.43)	129 (0.17)	129 (0.16)	
Other/unknown	258 (0.16)	325 (0.43)	363 (0.44)	

Data are expressed as number (%), or mean ± standard deviation. For categorical variables, we performed Chi-square tests, and for continuous variables, we performed *t*-tests to compare differences between groups.

ESKD, end-stage kidney disease; COVID-19, coronavirus disease 2019; REMC, regional emergency medical center; CEMC, community emergency medical center; CEMI, Community Emergency Medical Institute; KTAS, Korean Triage and Acuity Scale; ICU, intensive care unit; ED, emergency department.

Table 2 shows the top 10 diagnoses in the ED during both periods. The most common causes of ED visits were mechanical complications of other heart and vascular devices and implants (T825) in 2020-2021 and pneumonia (J189) in 2018-2019. However, the leading diagnoses for patients with ESKD visiting the ED showed no overall differences between the pre- and post-COVID-19 periods. When examining the leading causes of ED visits by hospital service level, the leading causes of ED visits varied by level of hospital service and differed at each center in both periods (Supplementary Table S1). Pulmonary edema and mechanical complications of other heart and vascular devices and implants were the leading causes at higher-level centers (REMCs and CEMCs, respectively), whereas diabetes mellitus with diabetic polyneuropathy was the leading cause at CEMIs during the COVID-19 pandemic.

**Table 2:** Top 10 primary diagnoses in ESKD patients who visited the emergency department before and during COVID-19.

Primary diagnosis	Diagnosis code by ICD-10^**a**^	ESKD 2018-2019	Primary diagnosis	Diagnosis code by ICD-10	ESKD 2020-2021
Pneumonia	J189	2481 (5.67)	Mechanical complication of other cardiac and vascular devices and implants	T825	2375 (4.71)
Pulmonary edema	J81	2036 (4.65)	Pneumonia	J189	2241 (4.44)
Mechanical complication of other cardiac and vascular devices and implants	T825	1947 (4.45)	Pulmonary edema	J81	2234 (4.43)
Dyspnea	R060	1410 (3.22)	Dyspnea	R060	1637 (3.25)
Hyperkalemia	E875	1275 (2.91)	Hyperkalemia	E875	1525 (3.02)
Gastrointestinal hemorrhage, unspecified	K922	1123 (2.56)	Gastrointestinal hemorrhage, unspecified	K922	1407 (2.79)
Fever	R509	990 (2.26)	Melena	K921	1005 (1.99)
Gastroenteritis and colitis of unspecified origin	A099	899 (2.05)	Gastroenteritis and colitis of unspecified origin	A099	844 (1.67)
Melena	K921	753 (1.72)	Heart failure, unspecified	I509	755 (1.50)
Heart failure, unspecified	I509	744 (1.70)	Fever	R509	697 (1.38)

Data are expressed as number (%).

ESKD, end-stage kidney disease; COVID-19, coronavirus disease 2019.

a The modified version of the International Classification of Diseases, 10th revision.

### Hospitalization and in-hospital mortality before and during COVID-19

**Figure 2:**
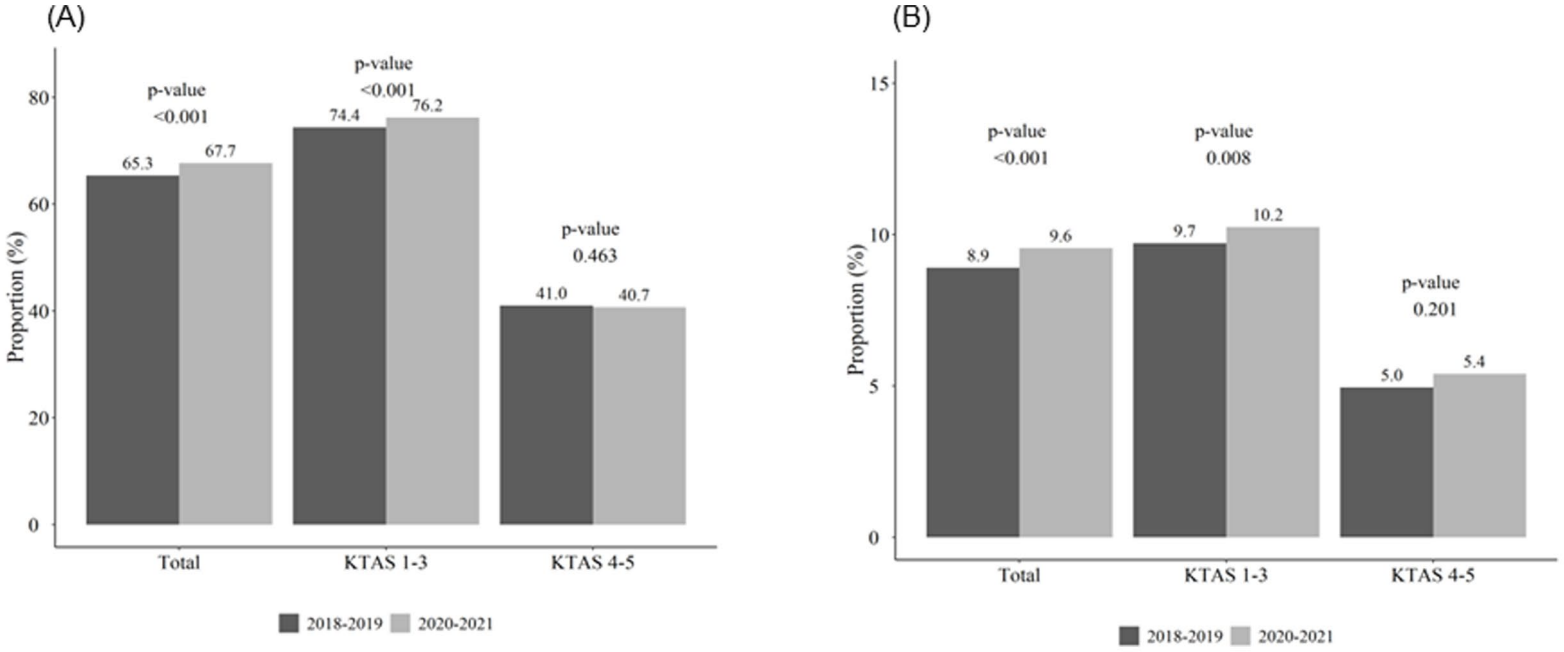
Hospitalization **(A)** and in-hospital mortality **(B)** rates according to KTAS classification. A Chi-square test was performed to compare differences between groups.

The rate of admission to a general ward or intensive care unit was higher in 2020–2021 than in 2018–2019, suggesting that fewer patients with ESKD were discharged during the COVID-19 pandemic. Figure 2 shows the hospitalization and in-hospital mortality rates according to KTAS classification. ED visits categorized as KTAS 1–3 showed higher rates of hospitalization and in-hospital mortality in 2020-2021 than in 2018-2019. However, for those with KTAS 4-5, no significant differences were observed between the two periods. Table 3 presents clinical outcomes of patients after hospital admission. In 2020-2021, the normal discharge and transfer rates decreased, whereas the voluntary and hopeless discharge rates increased. We conducted multivariate analyses to examine the differences in hospitalization and in-hospital mortality rates before and during COVID-19 (Supplementary Tables S2, S3). There were significantly higher ED hospitalization rate (odds ratio [OR]: 1.054; 95% confidence interval [CI]: 1.031–1.079; *p* < 0.001) and mortality rate (OR: 1.052; 95% CI: 1.007–1.099; *p* = 0.022) in 2020–2021 compared with 2018–2019.

**Table 3:** Comparison of clinical outcomes following emergency department before and during COVID-19.

Variables	Total	ESKD 2018-2019	ESKD 2020-2021	*p*-value
Number of admission	106,103	49,831	56,272	
Normal discharge	78,829 (74.29)	37,363 (74.98)	41,466 (73.69)	<0.001
Voluntary discharge	1795 (1.69)	695 (1.39)	1100 (1.95)	
Transfer	14,513 (13.68)	6837 (13.72)	7676 (13.64)	
Death	9811 (9.25)	4437 (8.90)	5374 (9.55)	
Escape	46 (0.04)	24 (0.05)	22 (0.04)	
Hopeless discharge	100 (0.09)	20 (0.04)	80 (0.14)	
Others	349 (0.33)	129 (0.26)	220 (0.39)	
Not available	660 (0.62)	326 (0.65)	334 (0.59)	

Data are expressed as number (%).

ESKD, end-stage kidney disease; COVID-19, coronavirus disease 2019.

### ESKD-related ED visits according to hospital service levels

In REMCs (Level 1), ED visits by ambulances and transfers from outpatient clinics were higher in 2020-2021 than in 2018-2019 (Table 4). In CEMCs (Level 2), the proportion of ED visits by patients aged ≥80 years was higher, and ED visits during the COVID-19 pandemic were more likely to be classified as emergency or urgent according to the KTAS classification. In CEMIs (Level 3), the proportion of ED visits by patients aged 30–39 and 40–49 years increased during the COVID-19 pandemic. ED visits by medical aid recipients, walk-ins, transport by ambulance, and KTAS 1 classification increased, whereas ED visits classified as KTAS 2–4. Non-emergency ED visits and discharges to home after an ED visit increased in CEMIs during the COVID-19 pandemic.

**Table 4:** Baseline characteristics of ESKD patients who visited the emergency department before and during COVID-19, by hospital service level.

Variables	REMC			CEMC			CEMI		
	Total	2018-2019	2020-2021	Total	2018-2019	2020-2021	Total	2018-2019	2020-2021
Number of ED visits	62,728	29,994	32,734	84,281	41,750	42,531	12,447	4,533	7,914
Age (years)									
20–29	938 (1.50)	447 (1.49)	491 (1.50)	828 (0.98)	390 (0.93)	438 (1.03)	52 (0.42)	22 (0.49)	30 (0.38)
30–39	2,261 (3.60)	1,084 (3.61)	1,177 (3.60)	2,485 (2.95)	1,264 (3.03)	1,221 (2.87)	597 (4.80)	147 (3.24)	450 (5.69)
40–49	5,325 (8.49)	2,743 (9.15)	2,582 (7.89)	6,822 (8.09)	3,616 (8.66)	3,206 (7.54)	894 (7.18)	325 (7.17)	569 (7.19)
50–59	11,771 (18.77)	6,055 (20.19)	5,716 (17.46)	15,029 (17.83)	7,726 (18.51)	7,303 (17.17)	2,192 (17.61)	875 (19.30)	1,317 (16.64)
60–69	16,328 (26.03)	7,650 (25.51)	8,678 (26.51)	21,518 (25.53)	10,692 (25.61)	10,826 (25.45)	3,178 (25.53)	1,257 (27.73)	1,921 (24.27)
70–79	16,126 (25.71)	7,672 (25.58)	8,454 (25.83)	22,618 (26.84)	11,376 (27.25)	11,242 (26.43)	3,421 (27.48)	1,206 (26.60)	2,215 (27.99)
≥80	9,979 (15.91)	4,343 (14.48)	5,636 (17.22)	14,981 (17.78)	6,686 (16.01)	8,295 (19.50)	2,113 (16.98)	701 (15.46)	1,412 (17.84)
Sex									
Men	35,981 (57.36)	17,079 (56.94)	18,902 (57.74)	48,167 (57.15)	23,696 (56.76)	24,471 (57.54)	7,428 (59.68)	2,703 (59.63)	4,725 (59.70)
Women	26,747 (42.64)	12,915 (43.06)	13,832 (42.26)	36,114 (42.85)	18,054 (43.24)	18,060 (42.46)	5,019 (40.32)	1,830 (40.37)	3,189 (40.30)
Insurance status									
National health insurance	47,646 (75.96)	22,632 (75.46)	25,014 (76.42)	64,379 (76.39)	31,814 (76.20)	32,565 (76.57)	8,636 (69.38)	3,228 (71.21)	5,408 (68.33)
Medical aid	14,563 (23.22)	7,108 (23.70)	7,455 (22.77)	19,063 (22.62)	9,569 (22.92)	9,494 (22.32)	3,507 (28.18)	1,231 (27.16)	2,276 (28.76)
Others	349 (0.56)	161 (0.54)	188 (0.57)	484 (0.57)	209 (0.50)	275 (0.65)	155 (1.25)	58 (1.28)	97 (1.23)
Uninsured	123 (0.20)	62 (0.21)	61 (0.19)	218 (0.26)	122 (0.29)	96 (0.23)	26 (0.21)	16 (0.35)	10 (0.13)
Unknown	47 (0.07)	31 (0.10)	16 (0.05)	137 (0.16)	36 (0.09)	101 (0.24)	123 (0.99)	0 (0.00)	123 (1.55)
Route of arrival									
Direct visit	38,479 (61.34)	18,305 (61.03)	20,174 (61.63)	57,103 (67.75)	28,298 (67.78)	28,805 (67.73)	10,006 (80.39)	3,576 (78.89)	6,430 (81.25)
Transferred from other hospital	18,282 (29.14)	9,016 (30.06)	9,266 (28.31)	20,157 (23.92)	10,334 (24.75)	9,823 (23.10)	1,938 (15.57)	773 (17.05)	1,165 (14.72)
Transferred from outpatient clinic	5,896 (9.40)	2,636 (8.79)	3,260 (9.96)	6,979 (8.28)	3,097 (7.42)	3,882 (9.13)	491 (3.94)	175 (3.86)	316 (3.99)
Other	69 (0.11)	36 (0.12)	33 (0.10)	42 (0.05)	21 (0.05)	21 (0.05)	12 (0.10)	9 (0.20)	3 (0.04)
Unknown	2 (0.00)	1 (0.00)	1 (0.00)	0 (0.00)	0 (0.00)	0 (0.00)	0 (0.00)	0 (0.00)	0 (0.00)
Transportation									
119 ambulance	15,487 (24.69)	7,079 (23.60)	8,408 (25.69)	22,536 (26.74)	10,858 (26.01)	11,678 (27.46)	3,444 (27.67)	1,174 (25.90)	2,270 (28.68)
Other ambulance	2,383 (3.80)	1,211 (4.04)	1,172 (3.58)	1,600 (1.90)	868 (2.08)	732 (1.72)	208 (1.67)	93 (2.05)	115 (1.45)
Non-ambulance	7,663 (12.22)	3,725 (12.42)	3,938 (12.03)	9,476 (11.24)	4,878 (11.68)	4,598 (10.81)	1,265 (10.16)	457 (10.08)	808 (10.21)
Unknown	37,195 (59.30)	17,979 (59.94)	19,216 (58.70)	50,669 (60.12)	25,146 (60.23)	25,523 (60.01)	7,530 (60.50)	2,809 (61.97)	4,721 (59.65)
KTAS classification									
1. Resuscitation	3,014 (4.80)	1,310 (4.37)	1,704 (5.21)	2,675 (3.17)	1,358 (3.25)	1,317 (3.10)	239 (1.92)	79 (1.74)	160 (2.02)
2. Emergent	9,986 (15.92)	4,594 (15.32)	5,392 (16.47)	12,358 (14.66)	5,791 (13.87)	6,567 (15.44)	1,352 (10.86)	528 (11.65)	824 (10.41)
3. Urgent	38,149 (60.82)	17,780 (59.28)	20,369 (62.23)	46,562 (55.25)	22,551 (54.01)	24,011 (56.46)	4,494 (36.11)	1,649 (36.38)	2,845 (35.95)
4. Less urgent	9,276 (14.79)	4,909 (16.37)	4,367 (13.34)	15,687 (18.61)	8,371 (20.05)	7,316 (17.20)	3,827 (30.75)	1,417 (31.26)	2,410 (30.45)
5. Non urgent	2,303 (3.67)	1,401 (4.67)	902 (2.76)	6,999 (8.30)	3,679 (8.81)	3,320 (7.81)	2,535 (20.37)	860 (18.97)	1,675 (21.17)
Length of stay	9.38 (11.01)	9.18 (11.41)	9.56 (10.63)	7.98 (10.04)	8.01 (10.88)	7.95 (9.14)	2.57 (2.94)	2.69 (2.94)	2.50 (2.93)
Disposition									
Discharge home	18,974 (30.25)	9,465 (31.56)	9,509 (29.05)	26,210 (31.10)	13,676 (32.76)	12,534 (29.47)	4,335 (34.83)	1,471 (32.45)	2,864 (36.19)
Transfer to other hospital	900 (1.43)	427 (1.42)	473 (1.44)	1,541 (1.83)	784 (1.88)	757 (1.78)	430 (3.45)	163 (3.60)	267 (3.37)
Admission									
General ward	31,871 (50.81)	15,115 (50.39)	16,756 (51.19)	41,752 (49.54)	20,333 (48.70)	21,419 (50.36)	5,702 (45.81)	2,216 (48.89)	3,486 (44.05)
ICU	10,589 (16.88)	4,798 (16.00)	5,791 (17.69)	14,295 (16.96)	6,717 (16.09)	7,578 (17.82)	1,894 (15.22)	652 (14.38)	1,242 (15.69)
Other	5 (0.01)	2 (0.01)	3 (0.01)	6 (0.01)	3 (0.01)	3 (0.01)	6 (0.05)	1 (0.02)	5 (0.06)
Died in ED	64 (0.10)	40 (0.13)	24 (0.07)	173 (0.21)	77 (0.18)	96 (0.23)	21 (0.17)	12 (0.26)	9 (0.11)
Other/unknown	325 (0.52)	147 (0.49)	178 (0.54)	304 (0.36)	160 (0.38)	144 (0.34)	59 (0.47)	18 (0.40)	41 (0.52)

Data are expressed as number (%), or mean ± standard deviation.

ESKD, end-stage kidney disease; COVID-19, coronavirus disease 2019; REMC, regional emergency medical center; CEMC, community emergency medical center; CEMI, Community Emergency Medical Institute; KTAS, Korean Triage and Acuity Scale; ICU, intensive care unit; ED, emergency department.

## Discussion

In this study, we analyzed the changes in ED utilization and clinical outcomes among patients with ESKD during the COVID-19 pandemic, when the number of ED visits, hospitalizations, and mortality rates of patients with ESKD after ED visits increased. Among patients with severe conditions, those classified as KTAS 1–3 at triage had higher rates of hospitalization and in-hospital mortality during COVID-19. There was a better distribution of ESKD-related ED visits by severity across different hospital service levels. ED visits classified as KTAS 1–3 at initial triage increased at higher-level centers (REMCs and CEMCs), while KTAS 2–4 decreased at CEMIs; for non-emergency ED visits, KTAS 5 increased.

As COVID-19 spread globally, the South Korean government raised the national infectious disease crisis alert level, mandated the wearing of masks, and recommended social distancing among individuals in public places. It also advised people not to visit a hospital immediately if they had respiratory symptoms or fever, and to self-isolate, undergo testing for COVID-19, and monitor their symptoms. As local or regional emergency centers began to struggle with overcrowding, patients with mild or tolerable conditions were advised to visit lower-level medical institutions or seek conservative care at home. Consequently, the number of ED visits during COVID-19 decreased, the proportion of younger patients decreased, and the proportion of older patients increased in South Korea (16). Patients with ESKD are often older and at high risk of mortality and morbidity from COVID-19 infection (17–20), resulting in an expected surge of ED visits and hospitalization rates during COVID-19 than in previous years.

ED utilization and related policies varied across countries during the COVID-19 pandemic. In Germany, the decline in hospitalizations varied among hospitals but was greater in the 0–17-year-old age group than in older age groups (21). Hospitalizations for all diagnoses except femur fracture and pneumonia declined, indicating delays in emergency care. In other countries, hospitalizations for severe conditions such as acute myocardial infarction, stroke, and trauma also declined significantly, though mortality rates were higher during the pandemic than before the pandemic (22–24). Na et al. examined the impact of the COVID-19 pandemic on hospitalization among patients with acute myocardial infarction, stroke, severe trauma, and excess mortality presenting to regional and local emergency medical centers in South Korea. They reported fewer ED visits and more ED deaths among patients with these major emergency conditions during the COVID-19 outbreak, which could mean that people with acute and life-threatening conditions avoided hospital care or delayed ED arrival or treatment.

In this study, the number of ESKD-related ED visits increased during the COVID-19 pandemic. Patients with ESKD who cannot fully self-isolate because of the need to visit dialysis centers three times a week are vulnerable to COVID-19. Additionally, dialysis units typically have high patient density, increasing the risk of infectious disease transmission. As a result, patients with ESKD may need to visit the ED more frequently than the general population to undergo testing for COVID-19 infection or other illnesses, even during the outbreak. Patients with ESKD visit EDs more frequently than the general population because of complications related to dialysis procedures or comorbidities (9, 10). When vascular access issues arise, hemodialysis cannot be performed. Treatment cannot be postponed because it may affect patient survival. Patients with ESKD may require ED visits. This was reflected in our findings, as vascular access issues were the leading cause of ED visits during COVID-19. Patients with ESKD who require dialysis in situations such as the COVID-19 outbreak may not be able to follow the same isolation and infection guidelines as the general population. Future guidelines for infection control and emergency systems should consider these characteristics.

In this study, the distribution of patients by hospital service level improved, with serious KTAS conditions (levels 1–3) increasing more frequently in REMCs. When comparing ED visits by hospital service level, patients who visited a CEMC or CEMI were less likely to be classified as KTAS levels 1-2 and more likely to have levels 3–5, while patients who visited an REMC were more likely to be classified as KTAS levels 1-2. Patients with less severe conditions who visited a CEMI were more likely to be evaluated and discharged from the ED without admission, whereas more severe patients tended to be concentrated in REMCs and the number of intensive care unit admissions increased. Vascular access issues were also a major cause of CEMC visits, as indicated by the improved distribution of severity across hospital service levels, with patients moving to lower-level centers such as CEMCs instead of REMCs. These findings demonstrate the enhanced role of the graded emergency medical system.

During the pandemic, patients with ESKD were classified as a high-risk population due to their need for regular dialysis center visits and their increased susceptibility to infection related to immunocompromised status. In addition to this vulnerability, ED visits were often unavoidable because of dialysis-related complications such as vascular access failure, hyperkalemia, and pulmonary edema, as well as other comorbidities. According to the findings of this study, vascular access complications, pulmonary edema, and hyperkalemia accounted for a substantial proportion of ED visits, and since these are preventable factors, early detection and proactive management of these issues may help reduce unnecessary ED utilization. Furthermore, because hospitalization and mortality were higher among patients classified as KTAS levels 1–3 at initial triage, appropriate allocation of patients to different hospital service levels based on disease severity is essential to prevent delays in treatment for critically ill patients. Notably, this study showed that severe cases were concentrated in higher level hospitals, indicating that the graded emergency medical system functioned effectively according to patient severity and institutional capacity. It will be necessary to develop a systematic scoring system to assess disease severity and determine the need for ED evaluation in vulnerable ESKD patients.

To the best of our knowledge, this is the first study to examine the impact of COVID-19 on ED utilization in the Korean emergency medical system among patients with ESKD. This study is significant because it is based on the National Emergency Department Information System (NEDIS), a highly reliable nationwide database in which all emergency medical organizations are required to participate. However, this study has several limitations. The dataset was administrative; while it was broad and population-based, it was lacked detailed individual-level comorbidity data. Additionally, we used ICD-10 code N18.5 to define patients with ESKD, meaning that we could not distinguish between dialysis modalities such as hemodialysis and peritoneal dialysis. Each record in the NEDIS database represents an ED visit rather than an individual patient; therefore, we were unable to account for repeated visits. Finally, we used ICD-10 diagnosis codes to identify primary conditions associated with ED visits, which may have resulted in misclassification due to coding bias and the inherent limitations of standardized coding systems.

In conclusion, the number of ESKD-related ED visits increased during the COVID-19 pandemic, likely due to the unique circumstances associated with dialysis. Hospitalization and in-hospital mortality following ED visits also increased in this patient population, particularly among patients with ESKD and severe conditions upon arrival at the ED. During the outbreak, patients with severe ESKD were more likely to present to higher-level EDs, and the distribution of patients across EDs improved.

## Data Availability

The raw data supporting the conclusions of this article will be made available by the authors, without undue reservation.

## Footnotes

1
https://www.R-project.org/
