# Supplementary material for: Emergency medical system utilization among patients with end-stage kidney disease during the COVID-19 pandemic: a retrospective cohort study in South Korea
Source: Front Public Health. 2026 Apr 7;14:1687521. doi: 10.3389/fpubh.2026.1687521 (PMC13095725; doi:10.3389/fpubh.2026.1687521)
Supplement: Supplementary file 1 [file Supplementary_file_1.docx]

**Table S1. Top 10 primary diagnoses in ESKD patients who visited emergency department by hospital service level**

| **REMC** | | | | | |
| --- | --- | --- | --- | --- | --- |
| Primary diagnosis | Diagnosis code by ICD-10^a^ | ESKD  2018-2019 | Primary diagnosis | Diagnosis code by ICD-10 | ESKD  2020-2021 |
| Pneumonia | J189 | 954 (5.56) | Pulmonary edema | J81 | 1,081 (5.4) |
| Pulmonary edema | J81 | 933 (5.52) | Mechanical complication of other cardiac and vascular devices and implants | T825 | 1,031 (5.15) |
| Mechanical complication of other cardiac and vascular devices and implants | T825 | 676 (4.0) | Pneumonia | J189 | 820 (4.09) |
| Hyperkalemia | E875 | 588 (3.48) | Hyperkalemia | E875 | 740 (3.7) |
| Gastrointestinal hemorrhage, unspecified | K922 | 458 (2.71) | Gastrointestinal hemorrhage, unspecified | K922 | 625 (3.12) |
| Dyspnea | R060 | 432 (2.56) | Dyspnea | R060 | 522 (2.61) |
| Fever | R509 | 365 (2.16) | Melena | K921 | 350 (2.25) |
| Melena | K921 | 318 (1.88) | Pleural effusion | J90 | 317 (1.58) |
| Gastroenteritis and colitis of unspecified origin | A099 | 309 (1.83) | Heart failure, unspecified | I509 | 312 (1.56) |
| Heart failure, unspecified | I509 | 294 (1.74) | Urinary tract infection | N390 | 308 (1.54) |
| **CEMC** |  |  |  |  |  |
| Primary diagnosis | Diagnosis code by ICD-10^a^ | ESKD  2018-2019 | Primary diagnosis | Diagnosis code by ICD-10 | ESKD  2020-2021 |
| Pneumonia | J189 | 1,403 (5.76) | Mechanical complication of other cardiac and vascular devices and implants | T825 | 1,341 (5.18) |
| Mechanical complication of other cardiac and vascular devices and implants | T825 | 1,263 (5.18) | Pneumonia | J189 | 1,241 (4.8) |
| Pulmonary edema | J81 | 1,010 (4.14) | Pulmonary edema | J81 | 1,002 (3.87) |
| Dyspnea | R060 | 809 (3.32) | Dyspnea | R060 | 898 (3.47) |
| Hyperkalemia | E875 | 646 (2.65) | Gastrointestinal hemorrhage, unspecified | K922 | 714 (2.76) |
| Gastrointestinal hemorrhage, unspecified | K922 | 632 (2.59) | Hyperkalemia | E875 | 706 (2.73) |
| Fever | R509 | 533 (2.19) | Melena | K921 | 527 (2.04) |
| Gastroenteritis and colitis of unspecified origin | A099 | 486 (1.99) | Gastroenteritis and colitis of unspecified origin | A099 | 415 (1.6) |
| Heart failure, unspecified | I509 | 422 (1.73) | Heart failure, unspecified | I509 | 396 (1.53) |
| Melena | K921 | 420 (1.72) | Chest pain | R074 | 360 (1.39) |
| **CEMI** |  |  |  |  |  |
| Primary diagnosis | Diagnosis code by ICD-10^a^ | ESKD  2018-2019 | Primary diagnosis | Diagnosis code by ICD-10 | ESKD  2020-2021 |
| Dyspnea | R060 | 169 (6.71) | DM with diabetic polyneuropathy | E14.42 | 232 (5.11) |
| Pneumonia | J189 | 124 (4.92) | Dyspnea | R060 | 217 (4.78) |
| Gastroenteritis and colitis of unspecified origin | A099 | 104 (4.13) | Pneumonia | J189 | 180 (3.96) |
| Pulmonary edema | J81 | 93 (3.69) | Pulmonary edema | J81 | 151 (3.32) |
| Fever | R509 | 92 (3.65) | Gastroenteritis and colitis of unspecified origin | A099 | 138 (3.04) |
| Dizziness | R42 | 57 (2.26) | Abdominal pain | R10.49 | 104 (2.29) |
| Chest pain | R074 | 47 (1.87) | Dizziness | R42 | 98 (2.16) |
| Abdominal pain | R10.49 | 46 (1.83) |  | R5099 | 94 (2.07) |
| Hyperkalemia | E875 | 41 (1.63) | Hyperkalemia | E875 | 79 (1.74) |
| Gastrointestinal hemorrhage, unspecified | K922 | 33 (1.31) | Fever | R509 | 74 (1.63) |

Data are expressed as number (%).

Abbreviations: ESKD, end-stage kidney disease; COVID-19, coronavirus disease 2019; REMC, regional emergency medical center; CEMC, community emergency medical center; CEMI, community emergency medical institute; DM, diabetes mellitus.

^a^The modified version of the International Classification of Diseases, 10th revision

**Table S2. Factors associated with hospitalization in ESKD patients who visited emergency department**

|  | | **Hospitalization** | |  | |
| --- | --- | --- | --- | --- | --- |
| **Variables** | | **OR (95% CI)** | | **p-value** | |
| Age (yr) | 20-29 | | 1 (reference) | |  |
|  | 30-39 | | 0.939 (0.837~1.054) | | 0.2896 |
|  | 40-49 | | 1.090 (0.980~1.213) | | 0.1133 |
|  | 50-59 | | 1.181 (1.065~1.309) | | 0.0016 |
|  | 60-69 | | 1.228 (1.108~1.360) | | <0.001 |
|  | 70-79 | | 1.356 (1.224~1.502) | | <0.001 |
|  | ≥80 | | 1.463 (1.318~1.624) | | <0.001 |
| Sex | Men | | 1 (reference) | |  |
|  | Women | | 0.970 (0.948~0.992) | | 0.0092 |
| Insurance status | National health insurance | | 1 (reference) | |  |
|  | Medical aid | | 0.862 (0.839~0.886) | | <0.001 |
|  | Others | | 1.383 (1.190~1.611) | | <0.001 |
|  | Uninsured | | 0.539 (0.430~0.678) | | <0.001 |
|  | Unknown | | 0.568 (0.445~0.725) | | <0.001 |
| Hospital level | I (REMC) | | 1 (reference) | |  |
|  | II (CEMC) | | 1.172 (1.144~1.201) | | <0.001 |
|  | III (CEMI) | | 1.649 (1.575~1.727) | | <0.001 |
| Route of arrival | Direct visit | | 1 (reference) | |  |
|  | Transferred from other hospital | | 1.652 (1.596~1.710) | | <0.001 |
|  | Transferred from outpatient clinic | | 1.603 (1.537~1.673) | | <0.001 |
|  | Other | | 1.039 (0.711~1.540) | | 0.844 |
|  | Unknown | | Not available | | 0.8138 |
| Transportation | 119 ambulance | | 1 (reference) | |  |
|  | Other ambulance | | 1.261 (1.147~1.388) | | <0.001 |
|  | Non-ambulance | | 1.536 (1.452~1.626) | | <0.001 |
|  | Unknown | | 0.672 (0.653~0.691) | | <0.001 |
| KTAS score | Level 1 | | 8.926 (8.218~9.704) | | <0.001 |
|  | Level 2 | | 7.893 (7.478~8.333) | | <0.001 |
|  | Level 3 | | 5.314 (5.086~5.552) | | <0.001 |
|  | Level 4 | | 1.720 (1.642~1.803) | | <0.001 |
|  | Level 5 | | 1 (reference) | |  |
| Length of stay |  | | 1.031 (1.030~1.033) | | <0.001 |
| Year | 2018-2019 | | 1 (reference) | |  |
|  | 2020-2021 | | 1.054 (1.031~1.079) | | <0.001 |

Abbreviations: ESKD, end-stage kidney disease; COVID-19, coronavirus disease 2019; REMC, regional emergency medical center; CEMC, community emergency medical center; CEMI, community emergency medical institute. Multivariate logistic regression analysis was conducted to assess the risk of hospitalization, adjusting for age group, gender, insurance status, hospital level, route of arrival, mode of transport, KTAS, length of stay, and the COVID-19 period.

**Table S3. Factors associated with mortality in ESKD patients who visited emergency department**

|  | | **Hospitalization** |  |
| --- | --- | --- | --- |
| **Variables** | | **OR (95% CI)** | **p-value** |
| Age (yr) | 20-29 | 1 (reference) |  |
|  | 30-39 | 2.044 (1.144~3.986) | 0.0233 |
|  | 40-49 | 2.981 (1.741~5.649) | <0.001 |
|  | 50-59 | 4.119 (2.428~7.754) | <0.001 |
|  | 60-69 | 5.456 (3.224~10.256) | <0.001 |
|  | 70-79 | 7.671 (4.535~14.412) | <0.001 |
|  | ≥80 | 10.547 (6.233~19.821) | <0.001 |
| Sex | Men | 1 (reference) |  |
|  | Women | 0.817 (0.781~0.854) | <0.001 |
| Insurance status | National health insurance | 1 (reference) |  |
|  | Medical aid | 0.933 (0.881~0.987) | 0.0165 |
|  | Others | 1.226 (0.957~1.551) | 0.0974 |
|  | Uninsured | 1.061 (0.687~1.581) | 0.7787 |
|  | Unknown | 1.072 (0.571~1.844) | 0.8147 |
| Hospital level | I (REMC) | 1 (reference) |  |
|  | II (CEMC) | 1.178 (1.125~1.233) | <0.001 |
|  | III (CEMI) | 1.098 (0.997~1.208) | 0.0569 |
| Route of arrival | Direct visit | 1 (reference) |  |
|  | Transferred from other hospital | 1.124 (1.049~1.204) | <0.001 |
|  | Transferred from outpatient clinic | 1.085 (0.979~1.199) | 0.1151 |
|  | Other | 1.336 (0.549~2.766) | 0.4755 |
|  | Unknown | Not available | 0.1556 |
| Transportation | 119 ambulance | 1 (reference) |  |
|  | Other ambulance | 1.621 (1.447~1.814) | <0.001 |
|  | Non-ambulance | 1.541 (1.421~1.670) | <0.001 |
|  | Unknown | 0.542 (0.511~0.574) | <0.001 |
| KTAS score | Level 1 | 6.446 (5.463~7.646) | <0.001 |
|  | Level 2 | 2.404 (2.054~2.830) | <0.001 |
|  | Level 3 | 1.472 (1.262~1.727) | <0.001 |
|  | Level 4 | 1.123 (0.948~1.335) | 0.1848 |
|  | Level 5 | 1 (reference) |  |
| Length of stay |  | 1.005 (1.003~1.006) | <0.001 |
| Year | 2018-2019 | 1 (reference) |  |
|  | 2020-2021 | 1.052 (1.007~1.099) | 0.0223 |

Abbreviations: ESKD, end-stage kidney disease; COVID-19, coronavirus disease 2019; REMC, regional emergency medical center; CEMC, community emergency medical center; CEMI, community emergency medical institute; KTAS, Korean Triage and Acuity Scale; OR odds ratio; CI, confidence interval. Multivariate logistic regression analysis was conducted to assess the mortality risk, adjusting for age group, gender, insurance status, hospital level, route of arrival, mode of transport, KTAS, length of stay, and the COVID-19 period.
